# Double promoter and tandem gene strategy for efficiently expressing recombinant FGF21

**DOI:** 10.1186/s12934-024-02447-5

**Published:** 2024-06-12

**Authors:** Longying Liu, Nuoyi Ning, Simeng Xu, Dongqing Chen, Luping Zhou, Zhimou Guo, Xinmiao Liang, Xianlong Ye

**Affiliations:** 1 Ganjiang Chinese Medicine Innovation Center, Nanchang, 330000 China; 2 Dalian Institute of Chemical Physics, Key Laboratory of Separation Science for Analytical Chemistry, Chinese Academy of Sciences, Zhongshan Road 457, Dalian, 116023 China

**Keywords:** Recombinant proteins, FGF21, Expression vector, Host cells, Double promoter, Tandem gene

## Abstract

**Background:**

Fibroblast growth factor 21 (FGF21) is a promising candidate for treating metabolic disorder diseases and has been used in phase II clinical trials. Currently, metabolic diseases are prevalent worldwide, underscoring the significant market potential of FGF21. Therefore, the production of FGF21 must be effectively improved to meet market demand.

**Results:**

Herein, to investigate the impact of vectors and host cells on FGF21 expression, we successfully engineered strains that exhibit a high yield of FGF21. Surprisingly, the data revealed that vectors with various copy numbers significantly impact the expression of FGF21, and the results showed a 4.35-fold increase in expression levels. Furthermore, the performance of the double promoter and tandem gene expression construction design surpassed that of the conventional construction method, with a maximum difference of 2.67 times.

**Conclusion:**

By exploring engineered vectors and host cells, we successfully achieved high-yield production of the FGF21 strain. This breakthrough lays a solid foundation for the future industrialization of FGF21. Additionally, FGF21 can be easily, quickly and efficiently expressed, providing a better tool and platform for the research and application of more recombinant proteins.

**Supplementary Information:**

The online version contains supplementary material available at 10.1186/s12934-024-02447-5.

## Background

Recombinant proteins have been a prominent area of research and application [1]. They have shown to be highly beneficial in key sectors, such as the biopharmaceutical, enzyme, and agricultural industries [2, 3], and have emerged as powerful weapons for combating previously untreatable diseases [4–8]. Microbial industrial fermentation serves as the primary source for the production of recombinant proteins, and the most utilized expression system is *Escherichia coli* [3, 9]. The market price of recombinant proteins is significantly influenced by the protein yield obtained, with notable price disparities between industrial and pharmaceutical applications; industrial proteins are priced in the range of tens of dollars per kilogram, while medical-grade proteins can reach billions of dollars per kilogram [1].

One potential medicinal protein candidate is FGF21, a peptide hormone that was discovered in 2000 and belongs to the endocrine FGF subfamily along with FGF23 and FGF15/19 [10]. FGF21 performs diverse metabolic functions [11], and multiple preclinical trials have demonstrated the potential of FGF21 in reducing plasma insulin levels [12–15]. In addition to its effects on insulin levels, FGF21 commonly leads to weight loss in animals [16–25]. In summary, due to the promising outcomes of these functional studies, numerous laboratories have actively pursued the development of recombinant FGF21 as a potential therapeutic option for patients with metabolic disorders, including obesity and diabetes.

The expression of recombinant FGF21 is predominantly achieved through prokaryotic expression systems. The expression strategy includes the fusion of various tags or modifications, such as SUMO or Fc fusion [26–29]. However, these strategies carry inherent risks. Pegazofermin and Efruxifermin, which are analogs of FGF21, have demonstrated long-term efficacy in treating nonalcoholic steatohepatitis through PEG (polyethylene glycol) modification and Fc fusion, respectively. These candidates have progressed to phase II clinical trials [30, 31]. Nonetheless, both candidates caused adverse reactions in clinical trials, such as mildly increased appetite, diarrhea, headache, and other symptoms.

Currently, achieving a high yield of recombinant FGF21 protein is challenging [26–32]. For instance, the fermentation of recombinant human FGF-21 (rhFGF-21) was performed in 30 L and 200 L fermenters by Hui Q et al. at the 200 L scale [32]. The final yield of purified FGF21 was only 71.1 ± 13.9 mg/L. Taken together, these results indicate that the traditional pET series of vectors are generally used for FGF21 expression, without any specific engineering or modification. The use of double promoter and tandem gene expression strategies in a prokaryotic expression system is a promising approach to increase yield and productivity [33–36]. In the present study, we initially explored expression vectors with different copy numbers. Furthermore, a synthetic biology-inspired design strategy involving multiple promoter tandem genes was employed to facilitate the expression of the target protein. Consequently, an engineered strain with a high yield of FGF21 was generated, providing an advantageous and useful method for the investigation and application of recombinant proteins.

## Methods

### Bacterial strains, plasmids, and reagents

The bacterial strains and plasmids used in this study are listed in Tables 1 and 2. The high, medium, and low copy number vectors pRSFDuet, pETDuet, and pACYCDuet, respectively, were purchased from Novagen (USA). The target fragments of seven double promoter and tandem gene constructs were synthesized by GenScript Biotechnology Co., Ltd. (Nanjing). The restriction enzymes, DNA polymerases, and T4 DNA ligase were purchased from Takara Biotechnology Co. (Japan). The commercial *E.coli* strain DH5α (Invitrogen, USA) was used for general cloning and plasmid maintenance, and *E.coli* BL21(DE3) and 6 other strains (Shanghai Weidi Biotechnology Co., Ltd.) were used as hosts for recombinant protein expression in this study. Other conventional reagents were purchased from Solarbio or Aladdin.

**Table 1:** Detailed information on the chemically competent cells

Strain names	Genotypes
BL21(DE3)	F- *omp* T *hsdSB*(r_B_^-^ m_B_^-^) *gal dcm* (DE3)
Rosetta(DE3)	F- *omp* T *hsdSB*(r_B_^-^ m_B_^-^) *gal dcm* (DE3) pRARE(*arg* U, *arg* W, *ilex*, *gly* T, *leu* W, *pro* L) (Cam^R^)
W3110(DE3)	F- λ- *rph-1* INV(*rrnD*, *rrnE*)(DE3)(Cam^R^)
BLR(DE3)pLysS	F-*omp* T *hsdSB* (r_B_^-^m_B_^-^) *gal dcm* (DE3) Δ(*srl-recA*)306:: Tn10 pLysS(Cam^R^, Tet^R^)
BL21(DE3)pLysS	F- *omp* T *hsdSB*(r_B_^-^ m_B_^-^) *gal dcm* (DE3)pLysS Cam^R^
Rosetta(DE3)pLysS	F- *omp* T *hsdSB*(r_B_^-^ m_B_^-^) *gal dcm* (DE3)pLysSRARE(Cam^R^)
ArcticExpress (DE3) pRARE2	*E. coli* BF- *ompThsdS* (r_B_^-^ m_B_^-^) *dcm*+ Tet^R^*gal * λ(DE3) *endA* Hte [*cpn10cpn60* Gent^R^] pRARE2 (Cam^R^)

### Construction of vectors for different strategies

As indicated in Table 2, vector construction involves the utilization of skeletons with varying copy numbers and different engineering strategies. There are three types vectors with different copy numbers. The pET vector is a medium-copy-number vector, as it carries the pBR322 replicator, which replicates approximately 20 to 40 copies in *E.coli*. There is also a high copy number vector called the pRSFDuet vector, which carries the RSF1030 replicator and achieves a replication number of approximately 100 replicas. On the other hand, the low-copy-number vector pACYCDuet can only replicate 10 to 12 copies due to the presence of the p15A replicator. In addition, a total of six different engineering strategies were designed, including the T7-P-P mode, in which a single promoter continuously activates two genes; the T7-T7-P-P mode, in which two promoters continuously activate a single gene; and the T7-P-T7-P mode, in which two promoters separately activate a single gene. The T7-P-P-T7-P mode includes two promoters and activates three genes separately, while the T7-P-P-T7-P-P mode double-activates and continuously activates four genes.

The engineered target fragments are usually synthesized by GenScript Biotechnology Co., Ltd., eliminating difficult self-construction processes, which is also essential for introducing synthetic biology design strategies. These synthetic gene fragments were inserted into different vectors which were contained by sphI and XhoI. These transformations with the constructed plasmids were carried out with the same batch of commercial *E.coli* DH5α chemocompetent cells according to a standard protocol. The transformants were selected and cultured in liquid. Furthermore, the plasmid was extracted and verified by enzyme digestion. Finally, the correct recombinant plasmid was obtained.

**Table 2:** Plasmids used in this study

Plasmids	Description	Source
pACYCDuet	Two T7 promoters, p15A replicon, Cm^R^, low copy number, 10 ~ 12 copies/cell	Novagen
pETDuet	Two T7 promoters, pBR322 replicon, Amp^R^, medium copy number, ~40 copies/cell	Novagen
pRSFDuet	Two T7 promoters, RSF1030 replicon, Kan^R^, high copy number, ~100 copies/cell	Novagen
FGF21/pACYCDuet	pACYCDuet containing FGF21 gene	This study
FGF21/pETDuet	pETDuet containing FGF21 gene	This study
FGF21/pRSFDuet	pRSFDuet containing FGF21 gene	This study
rhClp/pACYCDuet	pACYCDuet containing rhClp gene, highly repetitive gene	This study
rhClp/pETDuet	pETDuet containing rhClp gene, highly repetitive gene	This study
rhClp/pRSFDuet	pRSFDuet containing rhClp gene, highly repetitive gene	This study
T7-P	pETDuet containing FGF21 gene, The plasmid construction mode was T7-P	This study
T7-T7-P	pETDuet containing FGF21 gene, The plasmid construction mode was T7-T7-P	This study
T7-P-P	pETDuet containing FGF21 gene, The plasmid construction mode was T7-P-P	This study
T7-T7-P-P	pETDuet containing FGF21 gene, The plasmid construction mode was T7-T7-P-P	This study
T7-P-T7-P	pETDuet containing FGF21 gene, The plasmid construction mode was T7-P-T7-P	This study
T7-P-P-T7-P	pETDuet containing FGF21 gene, The plasmid construction mode was T7-P-P-T7-P	This study
T7-P-P-T7-P-P	pETDuet containing FGF21 gene, The plasmid construction mode was T7-P-P-T7-P-P	This study

### Expression and identification analysis

Recombinant expression plasmids with different copy number vectors were transformed into *E.coli* BL21(DE3) cells. Individual transformants picked separately were grown in 3 mL of Luria–Bertani (LB) medium supplemented with 100 µg/mL kanamycin at 37 °C. When the OD_600_ reached 0.6–0.8, IPTG was added to a final concentration of 0.25 mM. The culture was incubated sequentially at 37 °C for 4 h with shaking at 220 rpm. The cells were harvested and analyzed by sodium dodecyl sulfate‒polyacrylamide gel electrophoresis (SDS‒PAGE), and the expression level of the recombinant protein was detected by densitometry.

### Determination of growth profiles

As the expression of the strain was induced, the OD_600_ of the strain was measured every hour; that is, the growth profiles of the induced expression cells were monitored. The optical density of the suitably diluted samples was recorded at 600 nm with a Shimadzu UV2600 ultraviolet spectrophotometer (Japan). Finally, the software Origin was utilized to create a growth curve of the strain using the time and OD_600_ data, through which the strain’s growth trend and status were observed.

### Expression in different prokaryotic host cells

The recombinant FGF21/pETDuet (T7-P-P-T7-P-P) plasmid was transformed into seven different commercially available chemically competent cells (Table 1). BL21 (DE3) is a common recombinant protein expression cell line. The chromosome of this strain integrates the λ phage DE3 region and contains the T7 phage RNA polymerase, which is suitable for expressing genes cloned from expression vectors that contain the phage T7 promoter, such as the pET series. In the prokaryotic system, Rosetta (DE3) competent cells complement the tRNAs corresponding to six rare codons (AUA, AGG, AGA, CUA, CCC, GGA) lacking in *E.coli* to increase the expression level of foreign genes, especially eukaryotic genes. W3110(DE3) chemically competent cells can tolerate higher bacterial concentrations, increase the density of fermentation bacteria and increase protein expression. Chemically competent BLR (DE3)pLysS cells are suitable for expressing proteins encoded by genes containing unstable DNA or repetitive DNA sequences and for high-level expression of toxic and nontoxic proteins. Chemically competent BL21(DE3)pLysS and Rosetta(DE3)pLysS cells are suitable for high-level expression of toxic and nontoxic proteins. As the pLysSRARE plasmid can express the T7 lysozyme, which acts on the peptidoglycan on the cell wall to dissolve *E.coli*, the plasmid can also bind and inhibit the transcriptional activity of the T7 RNA polymerase, thereby reducing the background expression level of the target gene. ArcticExpress (DE3) pRARE2 chemically competent cells can express the chaperone proteins Cpn10 and Cpn60, which are adapted to low temperature, reduce the formation of inclusion bodies, and increase the expression and biological activity of soluble proteins.

The competent cells were thawed on ice, and then 100 ng of the recombinant plasmid was added to 50 µL of the competent cells. The mixture was thoroughly mixed, incubated on ice for 25 min, placed under heat shock at 42 °C for 45 s and immediately transferred to ice for 5 min. Then, 700 µL of LB medium was added to each tube containing the plasmid-transformed cells on a sterile workbench, and the cultures were incubated at 37 °C and 220 rpm for 1 h. Subsequently, 100 µL of the culture was spread onto LB plates containing the corresponding antibiotic marker, and the plates were incubated overnight at 37 °C until single colonies appeared.

### Quantification of recombinant proteins

The expression levels were determined from whole-cell samples collected at the end of harvest. All induced cells used in the study for expressing recombinant proteins were harvested upon completion of the experiment. Twenty milliliters of induced cells were centrifuged at 6,000 rpm at 4 °C for 10 min. The cell pellet was resuspended in 2 mL of PBS (pH 7.2). The induced cells were resuspended in SDS loading buffer and analyzed by 12% SDS‒PAGE. Equal volumes of the prepared samples were loaded onto SDS‒PAGE gels. Following sample migration, the gel was stained with Coomassie blue (Solarbio), after which the protein bands were visualized. Subsequently, the gel was washed with a washing gel solution to remove excess dye and improve the clarity of the stained bands. Images of the stained gel were captured using a Bio-Rad Molecular Imager® Gel Doc XR + Imaging Unit. The relative quantification of band intensity was estimated using Image Lab® software in the Bio-Rad Molecular Imager Gel Doc XR + unit by densitometric analysis [37].

### Large-scale fermentation

A frozen glycerol seed strain was inoculated into a 2 L flask that contained 400 mL of LB medium supplemented with 100 µg/mL kanamycin in a shaking incubator for approximately 20 h at 37 °C at 220 rpm. On the second day, the activated seed solution was transferred to 5 L fermenter medium at a ratio of 8:100 (v: v). When the OD_600_ reached 15.42, IPTG was added to a final concentration of 0.50 mM, and the mixture was incubated with shaking at 750 rpm at 30 °C. The fermentation medium consisted of 10 g/L glycerin, 16.3 g/L tryptone, 23 g/L yeast extract, 12 g/L NaCl, 1.31 g/L K_2_HPO_4_·3H_2_O, 2.5 g/L KH_2_PO_4_, 1.67 g/L (NH4)_2_SO_4_ and pH 7.2, with an initial working volume of 5 L. MgSO_4_·7H_2_O was sterilized separately and added to the fermentation medium at an initial concentration of 1 g/L. The agitation speed and flow rate of aeration were set at 300 rpm and 2.5 vvm (air volume/culture volume/min), respectively. The dissolved oxygen concentration was maintained above 30% of the air. During induction, glycerin was added at a constant low flow rate (180 mL/h), and the pH was stabilized at 7.0–7.2. After induction for 5 h, the cells were harvested by centrifugation at 19,500×g for 10 min at 10 °C, and the pellet was frozen at − 20 °C. During fermentation, the OD_600_ of the strain was measured every hour to monitor the growth profiles of the induced expression cells. Simultaneously, the expression of the recombinant protein was quantitatively analyzed by sampling at hourly intervals following induction.

### Purification of FGF21

All purification procedures were conducted under controlled temperature conditions at 4 °C. The frozen cell pellet was thawed and resuspended in ice-cold PBS buffer (pH 7.2) containing 0.5 M NaCl and 10,000 IU lysozyme for 20 g of cells. The cell suspension was sonicated in an ice bath. The insoluble pellet was collected by centrifugation at 12,000 rpm for 10 min. Subsequently, the sample was subjected to five washes with 35 mL of washing buffer each (20 mM Tris, 50 mM NaCl, 0.5 mM EDTA, 0.1% Triton X-100, pH 8.0). The washed precipitate was denatured at room temperature for 3 h using denaturing buffer (20 mM Tris, 8 M urea, 20 mM NaCl, 0.5 M EDTA, 2 mM DTT, pH 8.0). After denaturation, the protein was subjected to an overnight refolding process at 4 °C. The mixture was incubated in refolding buffer containing 20 mM Tris, 0.5 mM EDTA, 0.4 mM oxidized glutathione (GSH), 0.3 mM reductive glutathione (GSH), 0.02% Tween 80, and 10% glycerol at pH 8.0. The reduced protein samples were concentrated and replaced with 20 mM Tris, 1 mM EDTA, and 0.01% Tween 80 using a Labscale (Millipore). A Baby Bio Q ion-exchange chromatography column balanced with buffer A (20 mM Tris, 0.01% Tween, pH 8.0) was used to further purify the recombinant protein. Buffer B (20 mM Tris, 1 M NaCl, 0.01% Tween, pH 8.0) was eluted with equal amounts of 5% B, 20% B, and 100% B, and the target protein was obtained from the 20% B fraction. The concentration and purity of the target protein were determined using a BCA assay kit (Solarbio) and high-performance liquid chromatography (HPLC), respectively.

### Glucose uptake activity assay

At the logarithmic growth stage, Huh-7 cells (P7) were inoculated with 2 × 10^4^ cells (100 µL/well) in a 96-well plate with 3 replicates per group. The cells were incubated at 37 °C in a 5% CO_2_ incubator for 24 h. The culture medium was discarded, and the cells were starved for 24 h with serum-free DMEM. Positive and blank control wells were set and incubated at 37 °C in a 5% CO_2_ incubator for 24 h. Then, 100 µL of the protein to be measured was added to the experimental group at a final concentration of 2.5 µM, and 100 µL of the positive control group was added to the final concentration of 2.5 µM FGF21-S and FGF21-I purified by soluble expression and inclusion body expression, respectively, as described in previous studies [38]. An equal volume of PBS (pH 7.2) was added to the blank control groups. All samples were diluted with serum-free DMEM. At the end of the experiment, the GOD-POD method was used to detect the residual glucose content in the medium. A glucose test kit from Beijing Applygen Biotechnology Co., Ltd., was used.

## Results

### Construction of vectors for different strategies

**Fig. 1:**
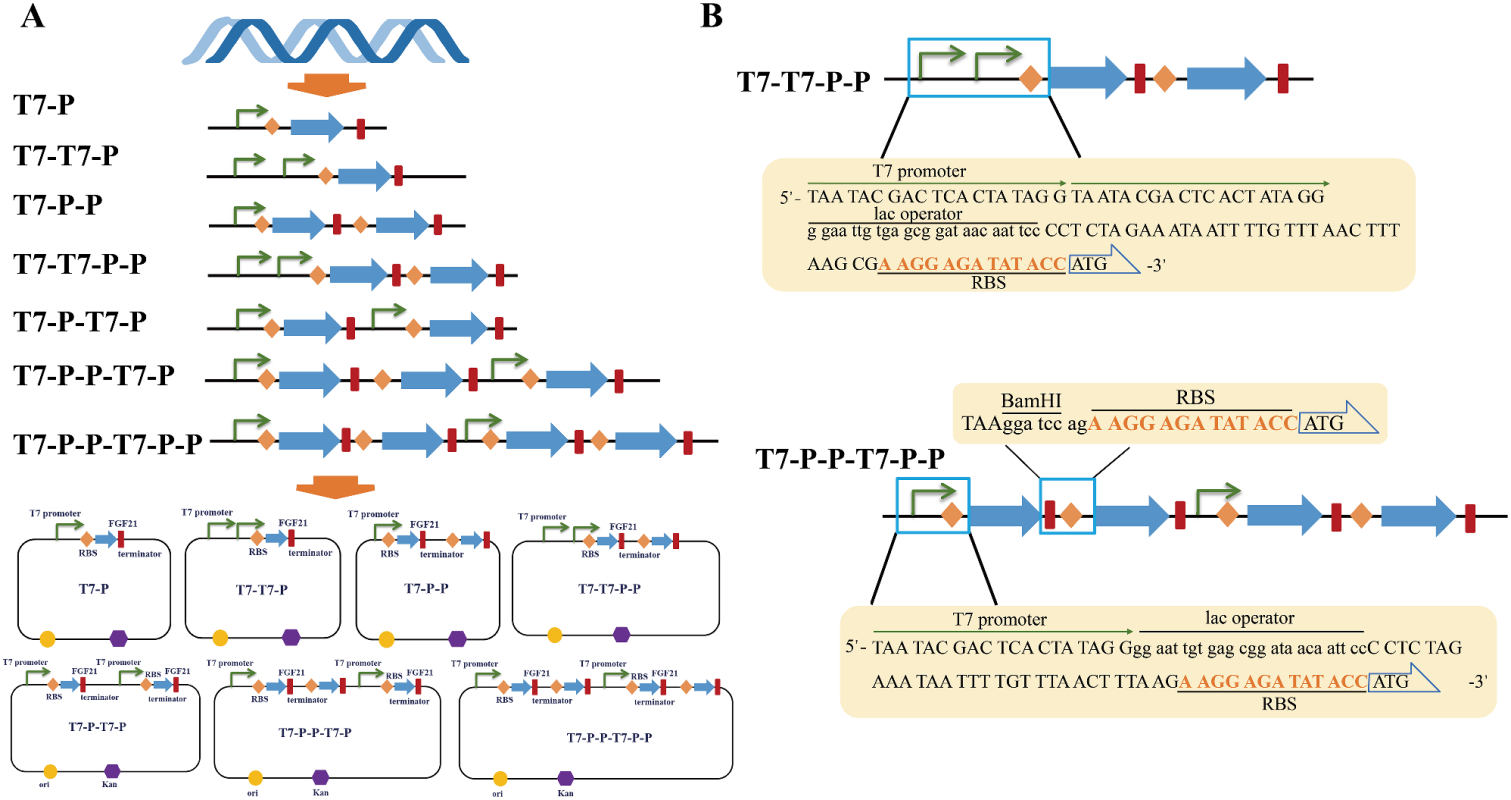
Construction of vectors for different strategies. **A** The plasmids used for different strategies in the construction of vectors. The design commences with DNA fragments and is subsequently inserted into the vector skeleton through gene synthesis connections, resulting in the formation of the ultimate recombinant plasmid. **B** Refined details of the strategy design. The T7 promoter, RBS, start codon of the target gene, and other element arrangement sequences and detailed sequence information were included

​To investigate the impact of different strategies on recombinant protein expression, we devised six different construction methods; the details are provided in Table 1; Fig. 1a. The pETDuet vector with a medium copy number was chosen as the skeleton for constructing the vector to avoid potential concerns related to high or low copy numbers. The design framework is depicted in Fig. 1b. Two promoters were constructed in tandem, T7-T7-P-P or T7-T7-P-P, in which two T7 promoters were connected in tandem, followed by a lac operator and a short cohesive sequence; in addition, the RBS region was placed before the ATG start codon of the target gene. The sequences and positions of all the elements refer to the original skeleton structure. Generally, the start codon to the end codon of the target gene follows the three-base codon translation principle to ensure the translation and expression without abnormality. The region from the promoter to the start codon may also be critical. Notably, the sequences of six different construction methods from the promoter to the start codon also adhere to the three-base codon translation principle.

Similarly, in the construction of multiple genes connected by double promoters, such as T7-P-P-T7-P-P, T7-P-P-T7-P, T7-P-T7-P, and T7-P-P, the sequence between the promoter and the target gene obeyed the 3-base codon translation principle. After considering how to concatenate a target gene after the first target gene, we added a restriction enzyme cut site after the first stopped codon in the target gene so that it could be removed if a problem arose. An RBS region was added immediately after the cleavage site. To comply with the three-base codon translation principle, two bases were added to the N-terminus of RBS, which were selected according to the GC content of the entire sequence. ​Following the same design ideas used to complete the various subsequent construction methods, GenScript ‘s gene synthesis helped achieve even more construction.

### Investigation to explore the impact of different copy number vectors on FGF21 expression

**Fig. 2:**
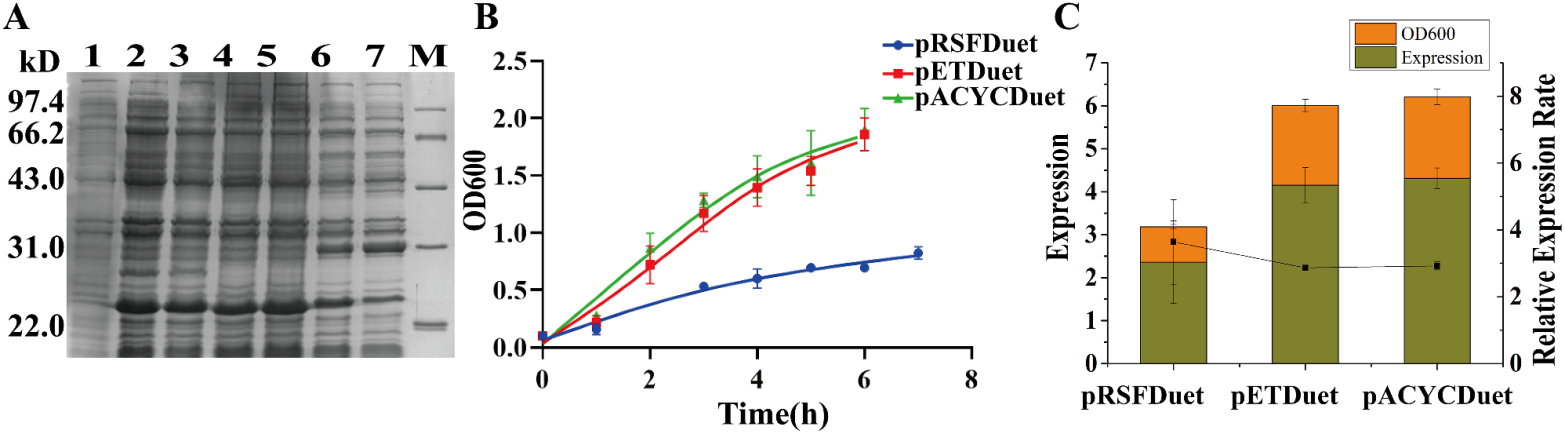
Expression level of recombinant FGF21 using vectors with different copy numbers. **A** 12% polyacrylamide gel analysis of cells expressing FGF21/pRSFDuet (high copy number, ∼100 copies/cell), FGF21/pETDuet (medium copy number, ∼40 copies/cell), or FGF21/pACYCDuet (low copy number, 10 ∼ 12 copies/cell). The cells were harvested at 37 ℃ and 100 rpm after 4 h of 0.25 mM IPTG induction. Lane 1: the whole cell without IPTG induction; Lanes 2/3: the whole cell with IPTG induction of the low-copy-number vector; Lanes 4/5: the whole cell with IPTG induction of the medium-copy-number vector; Lanes 6/7: the whole cell with IPTG induction of the high-copy-number vector; M:14.4–97.4 kDa protein marker. **B** Growth profiles of the cells expressing recombinant FGF21 with different copy number vectors. The OD_600_ of the collected bacterial fluid was measured at hourly intervals during expression induction. **C** Quantification analysis of the expression level of recombinant FGF21 in BL21(DE3) cells grown with different copy number vectors. The standard sample used for relative quantitative analysis of band intensity was Lane 7

The pET vector is a commonly used vector for the expression of recombinant proteins, but vector information has received little attention. To explore the influence of different copy number vectors, the expression levels of FGF21 were compared by transforming different copy number vectors into *E.coli*. As shown in Fig. 2a, the cells collected after induction were examined by 12% SDS‒PAGE. Compared with those in the noninduced sample, the high-, medium-, and low-copy-number vectors induced by IPTG exhibited obvious bands for target protein expression (lane 1). The expression levels of proteins in the high-copy-number vector samples were significantly lower, while those in the medium- and low-copy-number vector samples were greater.

During the induction of expression, OD_600_ data were collected to monitor the growth of the strains. The results are shown in Fig. 2b. Compared to the medium- and low-copy-number vectors, the high-copy-number vector exhibited noticeable growth retardation. Figure 2c presents the results obtained for the relative quantitative analysis of the expression outcomes for the three vectors. The histogram depicts the expression quantity and harvest OD_600_, while the dashed line represents the relative expression level. The results revealed that the three vectors with different copy numbers expressed the recombinant FGF21 protein, demonstrating a consistent trend in terms of expression amount, growth state, and relative expression level. In other words, there was no distinction between medium- and low-copy-number vectors, and both were significantly superior to the high-copy-number vector, with a maximum difference of 4.35 times.

Additionally, recombinant collagen (rhClp) with multiple sequence repeats was used as a target protein to explore the impact of different copy number vectors on gene expression. Unlike for FGF21, the high- and low-copy-number vectors were superior to the medium-copy-number vector, with a maximum difference of 5.85 times (supplementary data S1a/b/c). Therefore, for different recombinant proteins, the copy numbers of the expression vectors had varying effects and noticeable differences. Exploring the impact of expression vector copy numbers is extremely valuable.

### Interference of construction vectors for different strategies of FGF21 expression

**Fig. 3:**
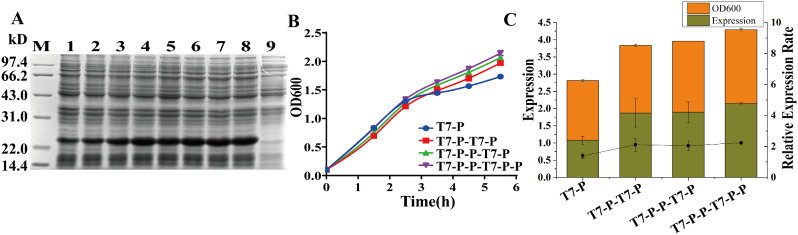
Expression level of the recombinant FGF21 protein using different vector construction strategies for double promoters and tandem genes. **A** Polyacrylamide gel analysis (12%) of FGF21/pETDuet (T7-P-T7-P), FGF21/pETDuet (T7-P-P-T7-P), and FGF21/pETDuet (T7-P-P-T7-P-P) cells. The cells were harvested at 37 ℃ and 100 rpm after 4 h of 0.25 mM IPTG induction. M: 14.4–97.4 kDa protein marker; Lanes 1/2: whole cell with IPTG induction of FGF21/pET30a(T7-P); Lanes 3/4: whole cell with IPTG induction of FGF21/pETDuet(T7-P-T7-P); Lanes 5/6: whole cell with IPTG induction of FGF21/pETDuet(T7-P-P-T7-P); Lanes 7/8: whole cell with IPTG induction of FGF21/pETDuet(T7-P-P-T7-P-P); Lane 9: whole cell without IPTG induction; **B** Growth profiles of the cells expressing recombinant FGF21 with different copy number vectors. The OD_600_ of the collected bacterial fluid was measured at hourly intervals during expression induction. **C** Quantification analysis of the expression level of recombinant FGF21 in BL21(DE3) cells grown with different copy number vectors. The standard sample used for relative quantitative analysis of band intensity was Lane 1

The expression levels of the FGF21 protein were compared by transforming the plasmids of different construction modes into *E.coli.* Figure 3a, Figure S2a, and Figure S2b show the expression of T7-T7-P1 (lanes 3/4) and T7-T7-P1-P1 (lanes 5/6). The uninduced sample is shown in lane 7. The supplementary data in Table S1 are the growth records of the strains expressed by the corresponding two construction models. These data showed that the continuous pattern of two T7 promoters continuously activating target genes did not affect the growth of the expressed strains; in addition, the pattern did not increase the expression level of the target protein and even decreased its expression. The results of the T7-P-P model were similar to those of the T7-T7-P and T7-T7-P-P models (supplementary data Table S2 and Figure S2b). Compared with that in the control T7-P model, the growth in the T7-P-P group did not significantly differ, and the expression level of T7-P-P was not significantly greater.

Figure 3a compares the expression of the T7-P-T7-P, T7-P-P-T7-P, and T7-P-P-T7-P-P strains in the constructed model with that of T7-P as the control using 12% SDS‒PAGE. The process involved collecting OD_600_ values of the strains during expression induction, thereby monitoring the strain’s growth, as depicted in Fig. 3b. Figure 3c illustrates the results obtained for the relative quantitative analysis of the expression outcomes from the three vectors. The histogram represents the expression quantity and harvest OD_600_, while the dashed line indicates the relative expression level. Figure 3a clearly illustrates that the three double promoter and tandem gene expression construction methods outperform the conventional expression construction method. The superiority of these double promoter and tandem gene series expression architecture patterns was evident and significant.

The results presented in Fig. 3b also demonstrated that the three double promoter and tandem gene series expression patterns did not adversely affect the growth of the expressed strains. A trend was observed in the growth patterns, with T7-P ≤ T7-P-T7-P-T7-P-P ≤ T7-P-T7-P-P ≤ T7-P-T7-P-P. This trend was consistent with the data obtained from the quantitative analysis, as shown in Fig. 3c. The collected data, including the harvest OD_600_, expression quantity, and relative expression level, further supported the trend of T7-P < T7-P-T7-P ≤ T7-P-P-T7-P-P ≤ T7-P-P-T7-P-P.Compared with that in the T7-P mode, the expression level in the T7-P-P-T7-P-P mode increased by a factor of 2.15, the harvest OD_600_ increased by 1.24 times, and the relative expression level increased by 1.61 times. Therefore, during a limited test in the experimental phase, there was a remarkable overall improvement of 2.67 times. Considering the 1.61-fold increase in relative expression levels, the fermentation platform has the potential to achieve higher expression levels. Remarkably, the three construction modes of double promoter and tandem gene expression significantly outperformed the regular T7-P construction mode.

### The suitability of host strains for FGF21 expression

**Fig. 4:**
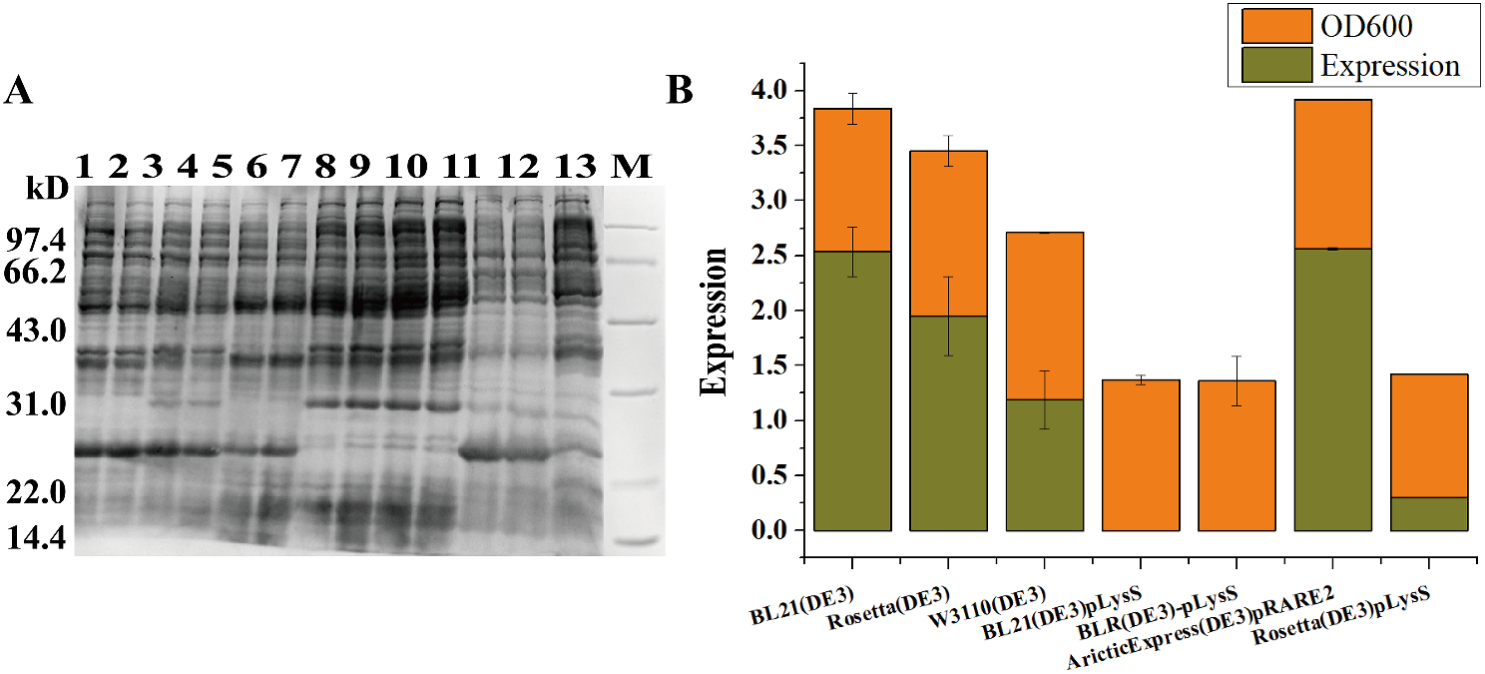
Expression levels of recombinant FGF21 protein in different prokaryotic expression cells. **A** Polyacrylamide gel (12%) analysis of cells with different prokaryotic expression levels. The cells were harvested at 37 ℃ and 100 rpm after 4 h of 0.25 mM IPTG induction. Lanes 1/2: whole cell with IPTG induction of BL21(DE3); Lanes 3/4: whole cell with IPTG induction of Rosetta(DE3); Lanes 5/6: whole cell with IPTG induction of W3110(DE3); Lanes 7/8: whole cell with IPTG induction of BL21(DE3)pLysS; Lanes 9/10: whole cell with IPTG induction of BLR(DE3)pLysS; Lanes 11/12: whole cell with IPTG induction of AricticExpress(DE3)pRARE2; Lanes 13: whole cell with IPTG induction of Rosetta(DE3) pLysS; M: 14.4–97.4 kDa protein marker; **B** Quantification analysis of the expression level of recombinant FGF21 in different prokaryotic expression cells. The standard sample used for relative quantitative analysis of band intensity was Lane 5

With advancements in biotechnology, a wide range of modified expression host strains are now commercially available. We purchased seven expression host strains for testing, as shown in Table 2. We transformed the optimized construction model FGF21/pETDuet (T7-P-P-T7-P-P) obtained by screening into seven different expression chassis to compare the effects. Similarly, the cells were collected and subjected to 12% SDS‒PAGE after culture at 37 ℃ and 100 rpm induced by 0.25 mM IPTG for 4 h, as shown in Fig. 4a and b. The preliminary visual evaluation showed a predominance of target protein expression in BL21(DE3) and ArcticExpress (DE3) pRARE2 cells; however, the target protein was almost not expressed in BLR(DE3)pLysS, BL21(DE3)pLysS or Rosetta(DE3)pLysS. The induced initial OD_600_ and harvest OD_600_ data are shown in Table 3. It can be concluded that the growth of the 7 expression strains was not abnormal, and the differences were not significant.

**Table 3:** The cellular density profiles of the cells expressing recombinant FGF21 protein with pETDuet (T7-P-P-T7-P-P)

Sample name	Induced initiation OD_600_	Harvest OD_600_
BL21(DE3)	0.61±0.01	1.49±0.23
Rosetta(DE3)	0.73±0.17	1.68±0.23
W3110(DE3)	0.86±0.08	1.80±0.42
BLR(DE3)pLysS	0.62±0.01	1.40±0.17
BL21(DE3)pLysS	0.73±0.04	1.55±0.23
Rosetta(DE3)pLysS	0.63±0.00	1.12±0.00
AricticExpress(DE3)pRARE2	0.61±0.03	1.61±0.29

Relative quantitative analysis was performed with the expression levels of different strains and combined with the harvest OD_600_ for summary evaluation, as shown in Fig. 4c. The results of the summary analysis indicated that the expression levels of seven types of chassis cells exhibited significant variations, and FGF21/pETDuet (T7-P-P-T7-P-P) exhibited the highest expression levels in both the BL21(DE3) and ArcticExpress(DE3)pRARE2 chassis. The difference in expression levels between these two chassis was minimal. Notably, the background expression level of ArcticExpress (DE3) pRARE2 was lower than that of BL21 (DE3).

### Fermentation (10 L) of the selected engineered FGF21 strain

**Fig. 5:**
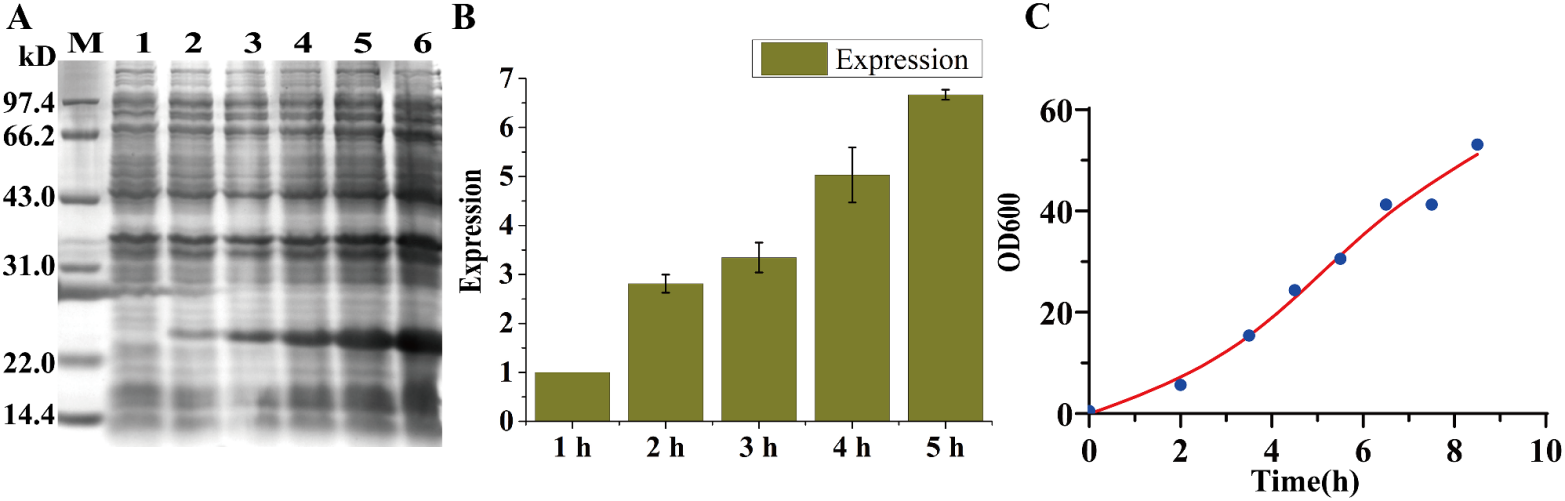
Expression levels of selected FGF21 engineered strains after 10 L of fermentation. **A** Polyacrylamide gel (12%) analysis of 10 L of fermentation in BL21(DE3) harboring FGF21/pETDuet (T7-P-P-T7-P-P). The cells were harvested at 37 ℃ and 100 rpm after 4 h of 0.25 mM IPTG induction. M: 14.4–97.4 kDa protein marker; Lane 1: whole cell without IPTG induction; Lanes 2/3/4/5/6: whole cell with IPTG induction of BL21(DE3) harboring FGF21/pETDuet (T7-P-P-T7-P-P). Samples were collected every hour during the induction period for subsequent analysis via 12% SDS‒PAGE. **B** Quantification analysis of the expression level of the BL21(DE3) strain harboring FGF21/pETDuet (T7-P-P-T7-P-P). The standard sample used for relative quantitative analysis of band intensity was Lane 2. **C** Growth profiles of the cells expressing recombinant FGF21 with 10 L of fermentation. The OD_600_ of the collected bacterial fluid was measured at hourly intervals during expression induction

To clarify the impact of the multipriming tandem expression mode on increasing the expression levels, a large-scale fermentation of 10 L was conducted using the dominant strain BL21(DE3) harboring FGF21/pET30a (T7-P-P-T7-P-P). The expression outcomes were measured and assessed accordingly. Detailed information regarding the specific fermentation parameters can be found in Table 4. When the OD_600_ reached 15.42, the experiment was induced with 0.25 mM IPTG. The induction was carried out at a temperature of 30 °C for 5 h. Samples were collected every hour during the induction period for subsequent analysis via 12% SDS‒PAGE (e.g., Fig. 5a). The experiment concluded when the OD_600_ reached 53.1. A total collection volume of 7.5 L was obtained, and 521 g of mud was collected through a tubular centrifuge.

**Table 4:** Data for the recombinant fermentation parameters

Fermentation parameter	Data information
Fermentation tank volume	10 L
Induced initiation OD_600_	15.42
Induction duration	5 h
Induction temperature	30 V
Harvest OD_600_	30 V
Harvest Fermentation fluid volume	7.50 L
Harvest Fermentation sludge	521g
Recombinant protein expression level	2.20 g/L

The expression of the recombinant protein continued to increase as the induction time increased. This trend was particularly evident in the bar chart depicted in Fig. 5b. Furthermore, as illustrated in Fig. 5c, the growth of the fermentation strain remained robust throughout the induction process, without reaching a growth plateau. Finally, a quantitative analysis using BCA detection revealed that the BL21(DE3)-expressing cells harboring FGF21/pET30a (T7-P-P-T7-P-P) generated a yield of 2.2 g/L protein after fermentation in a 10 L volume. These results indicate successful production of the target protein at a significant scale.

### Purification and bioactivity analysis of FGF21

**Fig. 6:**
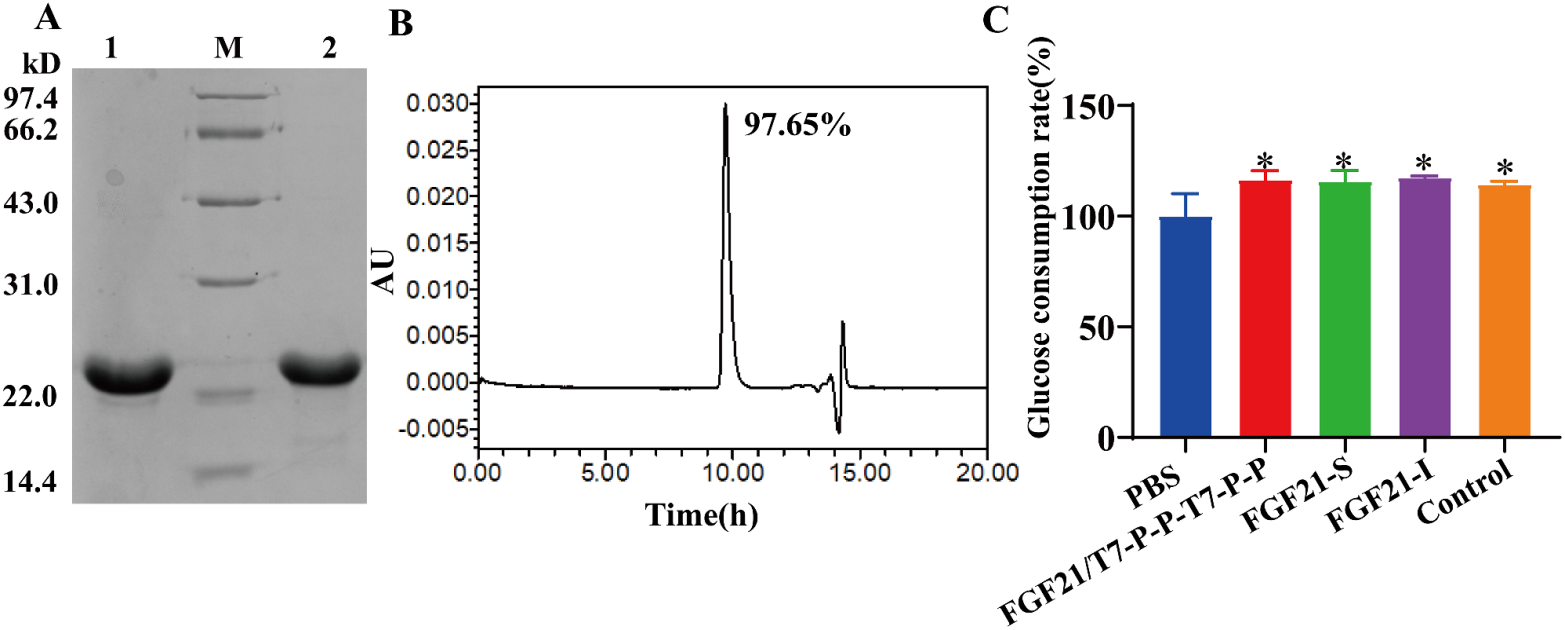
Purification and bioactivity analysis of recombinant FGF21. **A** The purified FGF21/pETDuet (T7-P-P-T7-P-P) was detected by 12% SDS‒PAGE. M: 14.4–97.4 kDa protein marker; Lane 1: Electrophoretic diagram of purified recombinant FGF21 under reducing conditions; Lane 2: Electrophoretic diagram of purified recombinant FGF21 under nonreducing conditions; **B** SEC-HPLC analysis of purified recombinant FGF21; **C** Bioactivity analysis of glucose consumption by purified recombinant FGF21. The FGF21/pETDuet (T7-P-P-T7-P-P) protein, or labeled as the FGF21/T7-P-P-T7-P-P protein, was purified by double promoter and tandem gene expression of the FGF21 protein in the form of an inclusion body, the FGF21-S protein was prepared by conventional construction and expressed in soluble form, and the FGF21-I protein was purified by conventional construction and expressed in the form of an inclusion body. Two-way ANOVA: *P < 0.05 vs. PBS. Dunnett’s multiple comparisons test. *N* = 3

The collected fermentation sludge was homogenized and broken, and the obtained inclusion body protein was denatured, renatured, and further purified by anion exchange chromatography. Ultimately, the recombinant FGF21 protein was successfully prepared, as shown in Fig. 6a. The results of reducing and nonreducing SDS‒PAGE analyses demonstrated that the purified recombinant FGF21 protein exhibited a monomeric structure. SEC-HPLC analysis revealed that the recombinant FGF21 was approximately 97.65% pure. The concentration of the protein, as determined by the BCA assay, was found to be 7.74 mg/mL. The yield was 591.44 mg/L, with a yield efficiency of 47.89%.

Next, the bioactivity of the recombinant FGF21 protein obtained through double promoter and tandem gene expression was evaluated. Three types of proteins, FGF21/T7-P-P-T7-P-P, FGF21-S, and FGF21-I, were used to stimulate Huh-7 cells after starvation treatment, and the glucose consumption rate was measured by the GOD-POD method 24 h later. As depicted in Fig. 6c, all three types of recombinant FGF21 proteins demonstrated glucose consumption activity in Huh-7 cells. Remarkably, the glucose consumption activity of the FGF21/T7-P-P-T7-P-P samples was consistent with that of the FGF21-S and FGF21-I samples. Therefore, the utilization of the double promoter and tandem gene expression formats did not impact the bioactivity of the FGF21 protein.

## Discussion

Nonalcoholic fatty liver disease (NAFLD) is a major category of metabolic disorder [39, 40] that presently impacts approximately a quarter of the global population, and the prevalence of NAFLD continues to increase as more people are affected by obesity [41–43]. FGF21 is considered a promising candidate for treating NAFLD [44, 45]. FGF21 research remains in the early stage, and the safety and efficacy of FGF21 have been evaluated in many preclinical trials, but the necessary doses are high [46–48]. The limited yield of recombinant FGF21 protein poses a challenge to its industrial production and application [30–32]. Therefore, the recombinant FGF21 protein must be produced in higher yields to meet the large market demand in the future. The development of synthetic biology and genetic engineering technology provides more possibilities for large-scale industrial applications of recombinant proteins.

The expression of FGF21 predominantly relies on the prokaryotic system, and the bacteriophage T7-protomer protein expression system is extensively utilized for the production of recombinant proteins in prokaryotes [49, 50]. Plasmids were initially proposed as gene cloning vectors in 1972 [51, 52]. To function as an effective expression system, plasmids should possess a functional and stable replicon to maintain the recombinant plasmids within the host system [53–55]. High copy numbers may facilitate the segregation of plasmids into daughter cells during cell division without the need for additional partitioning mechanisms. However, this high copy number places a metabolic burden on the host cell, thereby impacting overall cellular metabolism [56]. Conversely, plasmids with low copy numbers alleviate metabolic stress on host cells but carry a greater risk of instability during segregation [57]. Consequently, appropriate replicons must be carefully selected when designing protein expression systems [58]. In addition to the replicon, the expression of recombinant proteins involves various essential elements, including the T7 promoter, ribosome binding site sequence (RBS), target gene, terminator, and more. These elements serve as crucial design points that must be carefully considered during the construction of expression systems [59]. Various design strategies are available for arranging and combining these elements, and most of these strategies aim to increase gene expression levels within metabolic pathways [60–62]. These strategies are valuable for designing the expression of recombinant proteins and are worth exploring.

Here, we first explored the influence of different copy number vectors on the expression of recombinant FGF21 protein, and unexpectedly, the copy number of the vectors was strongly correlated with the expression of the recombinant protein. In most studies, the medium-copy-number vector is commonly utilized as the primary vector for the heterologous expression of recombinant proteins [45, 46]. Unfortunately, the impact of different copy number vectors on the expression of recombinant proteins is frequently disregarded or overlooked. Surprisingly, the results presented in this study revealed that the vector copy number has a significant influence on recombinant protein expression. This observation highlights the importance of considering multiple factors rather than relying on theoretical speculation. The data obtained from the current testing phase focused on two recombinant proteins: FGF21 and rhClp. From a sequence perspective, FGF21 is relatively common, whereas rhClp is characterized by its highly repetitive nature. These two proteins hold significant representative value and serve as typical examples in this context. This surprising finding reveals a very clear direction for the industrial application of more recombinant proteins to improve the expression level.

Furthermore, we achieved a notable increase in the expression of the recombinant FGF21 protein by employing various double promoter and tandem gene strategies. These strategies rely on synthetic biology design, and the designed DNA fragments are synthesized by GenScrip Biotechnology Co., Ltd. With other methods, completing the construction of the vectors successfully poses a challenge. Notably, this approach is simpler, faster, and easier to implement than complex cellular genetic engineering, which generates uncertain final results and is also cost-effective. Finally, we conducted a suitability screening of multiple expression cell lines and observed that the expression levels of recombinant FGF21 protein varied across different chassis. Through three aspects of research, we obtained high-yield strains engineered to produce FGF21.

Interestingly, during the evaluation of multiple construction strategies involving double promoters and tandem gene expression, the implementation of double T7 continuous priming (T7-T7-P, T7-T7-P-P) did not enhance the expression of the target protein. This finding suggested that the expression vector or chassis cells may not possess the capacity to handle the efficiency of double-promoter continuous priming effectively. In addition, the expression vector or chassis cell may lack the necessary capability to effectively withstand the event of sequential priming involving dual promoters. Similarly, the single promoter tandem multiple gene expression (T7-P-P) construct did not significantly improve the expression of the target protein, suggesting that a single T7 promoter could not effectively initiate the expression of two tandem target genes. In contrast, compared with the T7-P mode, the T7-P-T7-P, T7-P-P-T7-P, T7-P-P-T7-P-P, and T7-P-P-T7-P-P construction modes with double promoters and tandem gene expression markedly increased the expression of the recombinant FGF21 protein. To investigate the mechanism of changes in expression levels, we conducted a study on plasmid stability in the different construction modes. The results showed that the T7-P-P-T7-P-P mode has higher plasmid stability than the T7-P mode. It indicates that tandem and repetitive sequences of plasmid DNA do not lead to the instability of the plasmid. This may also be one of the factors that increase the expression of recombinant FGF21 (Data not shown). Taken together, these data unequivocally indicate that dual promoters are effective when activated individually. As the number of activated genes increases, the expression becomes stronger, which is reflected in the results of this paper.

Finally, recombinant FGF21 protein expression was performed in commercially available expression host cells with multiple functionalities. Interestingly, the expression of these genes varied across different host cells, showing inconsistency. A notable difference was often observed when the host cells either failed to express the desired protein or exhibited significantly low expression levels. Therefore, we speculate that commercial expression host cells usually carry plasmids, such as pLysSRARE, to confer specific functions, and these cells are more unstable. Moving forward, it becomes imperative to establish stable and efficient expression in host cells, which may require genome editing and engineering modifications. Importantly, a considerable investment of time and extensive experimentation will be necessary to achieve these objectives.

## Conclusions

The results of this study have significant guiding implications for increasing the quantity and efficiency of recombinant protein expression. Based on our speculation, we believe that the observed regularity in the current data may apply to partial recombinant proteins. However, a broader range of recombinant proteins should be tested to ensure the accuracy of our conclusions or to identify any potential special cases. This approach provides a more comprehensive understanding of the phenomenon investigated in this paper.

In conclusion, the present study assessed the various copy number vectors in the recombinant protein FGF21 expression system, as well as multiple construction modes involving tandem expression with multiple initiations. Additionally, the suitability of various expression chassis was examined. Notably, successful and efficient expression of the recombinant FGF21 protein was achieved. These findings have significant implications for the large-scale industrial production of the recombinant protein FGF21. Furthermore, this study proposed a straightforward and rapid design philosophy that can be easily implemented to attain high expression quantity and efficiency in the prokaryotic expression of more recombinant proteins.

### Electronic supplementary material

Below is the link to the electronic supplementary material.

Supplementary Material 1

## Data Availability

The datasets used or analyzed during the current study are available from the corresponding author upon reasonable request.
