# Supplementary material for: Double promoter and tandem gene strategy for efficiently expressing recombinant FGF21
Source: Microb Cell Fact. 2024 Jun 12;23:171. doi: 10.1186/s12934-024-02447-5 (PMC11167883; doi:10.1186/s12934-024-02447-5)
Supplement: Supplementary file 1 — Supplementary Material 1 [file 12934_2024_2447_MOESM1_ESM.docx]

**SUPPLEMENTARY MATERIAL**

**Double promoter and tandem gene strategy for efficiently expressing recombinant FGF21**

*Longying Liu^1^, Nuoyi Ning^1^, Simeng Xu^1^, Dongqing Chen^1^, Luping Zhou^1^, Zhimou Guo^1,2^, Xinmiao Liang^1,2*^, Xianlong Ye ^1*^*

1. Ganjiang Chinese Medicine Innovation Center, Nanchang 330000, China

2. Dalian Institute of Chemical Physics, Chinese Academy of Sciences, Key Laboratory of Separation Science for Analytical Chemistry, Zhongshan Road 457, Dalian 116023, China

* Correspondence should be addressed:

[XY](mailto:XY) (yexianlong@jcmsc.cn), and XL (liangxinmiao@jcmsc.cn).


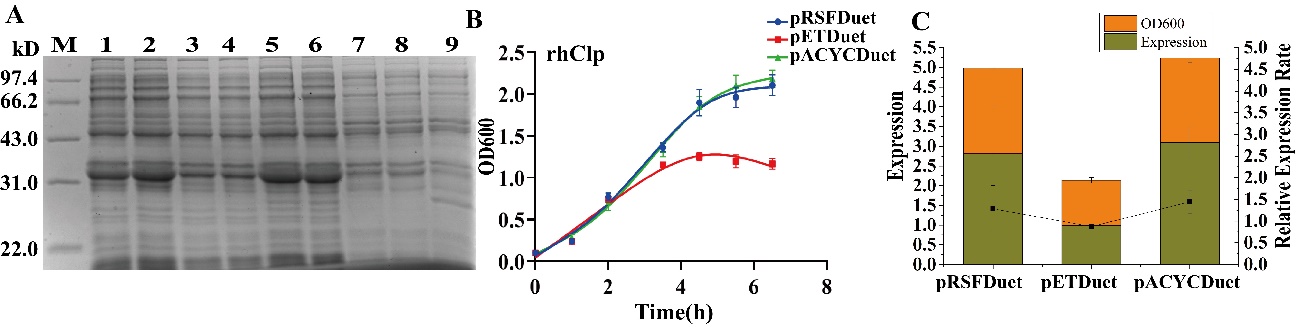


**Fig. S1 The expression level of recombinant collagen using vectors with different copy numbers. A** Twelve percent polyacrylamide gel analysis of cells expressing rhClp/pRSFDuet (high copy number, ∼100 copies/cell), hClp/pETDuet (medium copy number, ∼40 copies/cell), or rhClp/pACYCDuet (low copy number, 10 ∼ 12 copies/cell) was performed. The cells were harvested at 37 ℃ and 100 rpm after 4 h of 0.25 mM IPTG induction. M: 14.4–97.4 kDa protein marker; Lanes 1/2: whole cell with IPTG induction of the high-copy-number vector; Lanes 3/4: whole cell with IPTG induction of the medium-copy-number vector; Lanes 5/6: whole cell with IPTG induction of the low-copy-number vector; Lanes 7/8/9: whole cell without IPTG induction of the high-, medium-, and low-copy-number vectors; **B** Growth profiles of the cells expressing rhClp with vectors of different copy numbers. The OD_600_ of the collected bacterial fluid was measured at hourly intervals during expression induction. **C** Quantification analysis of the expression level of recombinant rhClp in BL21(DE3) cells grown with vectors of different copy numbers. The standard sample used for relative quantitative analysis of band intensity was Lane 3.


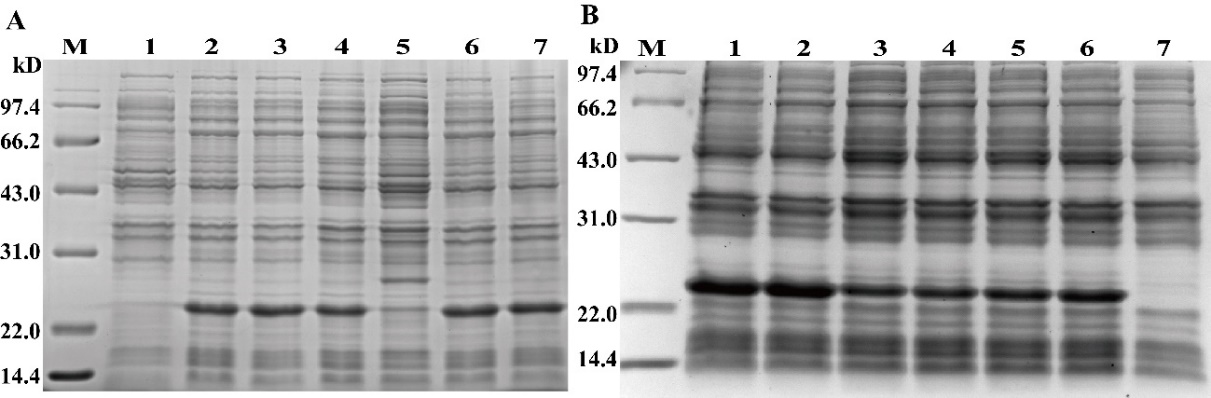


**Fig. S2 The expression level of recombinant FGF21 using vectors with different vector construction strategies.A** Twelve percent polyacrylamide gel analysis of FGF21/pETDuet (T7-T7-P)- and FGF21/pETDuet (T7-T7-P-P)-expressing cells. The cells were harvested at 37 ℃ and 100 rpm after 4 h of 0.25 mM IPTG induction. M: 14.4–97.4 kDa protein marker; Lanes 1/2: whole cell with IPTG induction of FGF21/pETDuet(T7-P); Lanes 3/4: whole cell with IPTG induction of FGF21/pETDuet(T7-T7-P); Lanes 5/6: whole cell with IPTG induction of FGF21/pETDuet(T7-T7-P-P); Lane 7: whole cell without IPTG induction; **B** Twelve percent polyacrylamide gel analysis of FGF21/pETDuet (T7-T7-P)- and FGF21/pETDuet (T7-P-P)-expressing cells. M: 14.4–97.4 kDa protein marker; Lane 1: whole cell without IPTG induction; Lanes 2/3: whole cell with IPTG induction of FGF21/pETDuet(T7-P); Lanes 4/5: whole cell with IPTG induction of FGF21/pETDuet(T7-T7-P); Lanes 6/7: whole cell with IPTG induction of FGF21/pETDuet(T7-P-P).

**Table S1. The growth records of the strains expressed by the corresponding two construction models of FGF21/pETDuet (T7-T7-P) and FGF21/pETDuet (T7-T7-P-P).**

**Table S2. The growth records of the strains expressed by the corresponding two construction models of FGF21/pETDuet (T7-T7-P) and FGF21/pETDuet (T7-P-P).**
